# ^18^F-Fluorocholine PET/CT Compared with Current Imaging Procedures for Preoperative Localization of Hyperfunctioning Parathyroids in Patients with Chronic Kidney Disease

**DOI:** 10.3390/diagnostics13081374

**Published:** 2023-04-08

**Authors:** Samuel Aymard, Benjamin Leroy-Freschini, Ashjan Kaseb, David Marx, Mehdi Helali, Gerlinde Averous, Valérie Betz, Sophie Riehm, Michel Vix, Peggy Perrin, Alessio Imperiale

**Affiliations:** 1 Department of Nuclear Medicine and Molecular Imaging, Institut de Cancérologie de Strasbourg Europe (ICANS), University of Strasbourg, 67200 Strasbourg, France; 2 Department of Radiology, College of Medicine, University of Jeddah, Jeddah 23890, Saudi Arabia; 3 Department of Medicine C, Hôpital de Sélestat, 67600 Sélestat, France; 4 Department of Nephrology and Transplantation, Strasbourg University Hospitals, University of Strasbourg, 67000 Strasbourg, France; 5 Department of Pathology, Strasbourg University Hospitals, University of Strasbourg, 67000 Strasbourg, France; 6 Department of Nephology, Hôpital de Colmar, 68024 Colmar, France; 7 AURAL Dialysis Center, 68000 Colmar, France; 8 Department of Radiology, Strasbourg University Hospitals, 67098 Strasbourg, France; 9 Department of General, Digestive, and Endocrine Surgery, IRCAD-IHU, Strasbourg University Hospitals, 67000 Strasbourg, France; 10 Molecular Imaging—DRHIM, IPHC, UMR 7178, CNRS, University of Strasbourg, 67093 Strasbourg, France

**Keywords:** hyperparathyroidism, secondary hyperparathyroidism, tertiary hyperparathyroidism, chronic kidney disease, parathyroid, ^18^F-fluorocholine, PET/CT, parathyroid scintigraphy, parathyroid CT, 4D-CT

## Abstract

Hyperparathyroidism (HPT) in patients with chronic kidney disease (CKD) includes secondary (sHPT) and tertiary hyperparathyroidism (tHPT). Considering that the role of preoperative imaging in the clinical setting is controversial, in the present study we have retrospectively compared pre-surgical diagnostic performances of ^18^F-Fluorocholine (^18^F-FCH) PET/CT, cervical ultrasonography (US), parathyroid scintigraphy, and 4D-CT in a group of 30 patients with CKD and HPT (18/12 sHPT/tHPT), 21 CKD G5 including 18 in dialysis, and 9 kidney transplant recipients. All patients underwent ^18^F-FCH, and 22 had cervical US, 12 had parathyroid scintigraphy, and 11 had 4D-CT. Histopathology was the gold standard. Seventy-four parathyroids were removed: 65 hyperplasia, 6 adenomas, and 3 normal glands. In the whole population, in a *per gland* analysis, ^18^F-FCH PET/CT was significantly more sensitive and accurate (72%, 71%) than neck US (25%, 43%), parathyroid scintigraphy (35%, 47%), and 4D-CT (40%, 47%). The specificity of ^18^F-FCH PET/CT (69%) was lower than that of neck US (95%) and parathyroid scintigraphy (90%), without, however, achieving significance. ^18^F-FCH PET/CT was more accurate than all other diagnostic techniques when sHPT and tHPT patients were considered separately. ^18^F-FCH PET/CT sensitivity was significantly higher in tHPT (88%) than in sHPT (66%). Three ectopic hyperfunctioning glands (in three different patients) were all detected by ^18^F-FCH PET/CT, two by parathyroid scintigraphy, and none by cervical US and 4D-CT. Our study confirms that ^18^F-FCH PET/CT is an effective preoperative imaging option in patients with CKD and HPT. These findings may be of greater importance in patients with tHPT (who could benefit from minimally invasive parathyroidectomy) than in patients with sHPT, who often undergo bilateral cervicotomy. In these cases, preoperative ^18^F-FCH PET/CT may be helpful in locating ectopic glands and may guide the surgical choice for gland preservation.

## 1. Introduction

Hyperparathyroidism (HPT) in patients with chronic kidney disease (CKD) includes secondary (sHPT) and tertiary hyperparathyroidism (tHPT). While the underling disease processes are related, sHPT and tHPT are two distinct and separate entities. By definition [1], tHPT is preceded by a prolonged period of sHPT and can be distinguished from sHPT by the presence of hypercalcemia in contrast to hypocalcemia in sHPT. With a successful kidney transplantation, metabolic and biochemical disturbances will be corrected, leading to spontaneous resolution of HPT in about 60% of patients [2,3].

Secondary to chronic kidney disease, the parathyroid glands develop glandular hypertrophy, resulting in parathormone (PTH) hypersecretion (sHPT). Kidney transplantation restores GFR and corrects mineral disorders with regression of parathyroid hyperplasia within several months after transplantation. However, in 20–30% of patients, one or several glands may become autonomous with persisting elevated levels of PTH, leading to hypercalcemia and hypophosphatemia. This would result in an increase in morbidity and mortality, mostly related to cardiovascular events, bone-related events (osteopenia, bone pain, pathologic fractures), vascular calcifications, severe pruritis, and nephrocalcinosis [1,4,5].

In patients suffering from CKD and HPT, the choice between a medical and a surgical treatment has yet to be standardized [6]. In daily practice, calcimimetics, calcitriol, and vitamin D analogues are widely used as a first-line therapy to lower PTH secretion. Parathyroidectomy (PTX) must be considered, particularly in patients with advanced CKD and severe biochemical or clinical sHPT refractory to medical management [7,8,9]. Multiglandular disease is common and the cure rate as well as the surgery-related complication rate after PTX are variable as they depend both on the patient’s comorbidities and on the chosen surgical approach [10,11,12]. Furthermore, once a patient is scheduled for surgery, the presence of ectopic parathyroid tissue, supernumerary glands, or aberrant parathyroid tissue from previous neck surgery must be preoperatively assessed [13].

The main objective of parathyroid imaging is to detect all sites with pathologic parathyroid hormone secretion prior to surgery. Over the years, several imaging techniques have been considered and utilized for this [14,15,16]. In a preoperative setting, neck ultrasonography (US), eventually coupled with 99mTc-sestamibi parathyroid scintigraphy, is considered the first-line imaging procedure, although it often shows suboptimal results in multi-gland related HPT [17,18]. US diagnostic performance is directly correlated with the experience of the operator, and the success of scintigraphy largely depends on the adopted scintigraphic protocol [19,20] as well as on the patient’s underlying pathology. Accordingly, the systematic review by Ruda et al. [21] highlights the low sensitivity of 99mTc-sestamibi scintigraphy in the detection of multi-gland hyperplasia (44%) and double adenomas (29.9%). In recent years, positron emission tomography/computed tomography (PET/CT) with 18F-fluorocholine (18F-FCH) has emerged as a second-line imaging procedure which offers high resolution, low radiation exposure in comparison with 4D CT, and shorter examination times in patients with primary HPT (pHPT), and even in patients with a history of PTX [22,23,24,25,26]. Unlike what has previously been published for pHPT, informative data on the use of 18F-FCH PET/CT in patients with CKD and HPT are limited [27,28]. Moreover, patients are usually analyzed without clear separation between sHPT and tHPT, which seems to be a questionable methodological choice when considering the underlying different pathophysiology. Thus, in the present study, we have evaluated the diagnostic performance of 18F-FCH PET/CT in patients with sHPT and tHPT scheduled for parathyroidectomy, and then compared it with the available pre-surgical conventional imaging procedures.

## 2. Materials and Methods

### 2.1. Patient Population

This is a retrospective, non-interventional, single-center study which includes dialysis patients and kidney transplant recipients with HPT who were referred to our nuclear medicine department for 18F-FCH PET/CT between January 2019 and December 2021. Only patients satisfying the following criteria were included: (1) sHPT or tHPT, distinguished by the calcium level: normo or hypocalcemia in sHPT and hypercalcemia in tHPT (hypercalcemia was assessed before introduction of calcimimetic) [1], (2) at least one additional pre-surgical procedure such as neck ultrasonography, parathyroid scintigraphy, or contrast-enhanced CT, or (3) parathyroid surgery [29] achieved after 18F-FCH PET/CT.

The following data were collected, retrospectively: (a) clinical symptoms, (b) measurements of serum calcium, phosphorus, intact PTH, and creatinine values, (c) patient’s genetic status, (d) first-line imaging including at least one examination amongst neck ultrasonography (US), 99mTc-sestamibi parathyroid scintigraphy, and contrast-enhanced CT, (e) pharmacological treatment of HPT at the time of PET/CT (if any), (f) surgical procedure, and (g) histological findings after parathyroidectomy. Both clinical and biochemical follow-ups of at least 3 months post-surgery were obtained for all patients, including serum PTH and calcium levels. A cross-disciplinary team described 18F-FCH PET/CT indications. Patient management was discussed on a case-by-case basis based on the clinical and surgical investigators involved in the decision-making process. In accordance with local institutional guidelines, all patients included gave a free and written informed consent for the use of the anonymous personal medical data extracted from their file for scientific or epidemiological purposes. The local Institutional Review Board approved this retrospective study (CE-2022-64).

### 2.2. Parathyroid Imaging

Parathyroid US was performed using a high-frequency linear transducer with the patient in a supine position and a hyperextended neck. Cross-sectional and longitudinal images of the neck and from the level of the carotid bifurcation to the superior mediastinum have been performed by an expert radiologist. Hypoechoic nodules of parathyroid location and echo structure different from that of a lymph node were considered positive on cervical ultrasound. The size and location of suspected parathyroid glands were also reported.

All scintigraphic and PET/CT procedures were achieved according to the EANM- practice guidelines for parathyroid imaging [19]. Parathyroid scintigraphy was performed using a dual-tracer subtraction technique (99mTc-sestamibi/123I). Imaging protocol included neck and mediastinum anterior planar scan, followed by single-photon emission tomography/computed tomography (SPECT/CT) acquisition. The CT scan was performed as a low-dose scan exclusively for anatomical correlation. The number of pathological findings, topography in reference to the thyroid gland, and ectopic locations were all recorded.

18F-FCH PET/CT was performed using a combined PET/CT device with time-of-flight measurement capability (Biograph128 mCT or Biograph Vision 600, Siemens Healthcare, Erlanger, Germany). Patients fasted for at least 6 h before the administration of 18F-FCH. Standard acquisition was performed 60 min (+/−5 min) after intravenous injection of 2–3 MBq/kg of 18F-FCH. All data were acquired on patients in the supine position with headrest and their arms along their body, from the mandible to the carina, with a PET duration time of 10–15 min. PET datasets were reconstructed iteratively using non-contrast CT for attenuation correction (OSEM algorithm). Attenuation-corrected and non-enhanced PET/CT readings including visual interpretation and measurement of quantitative parameters were performed centrally on a dedicated workstation (syngo.via VB30, Siemens Healthcare, Erlanger, Germany). PET/CT images were independently interpreted by two experienced nuclear medicine physicians. In cases of conflicting results between the two reviewers, a consensus was reached. The physicians were aware of the patients’ clinical data and biological investigations, but not of the results of first-line parathyroid imaging. Any focal non-physiological uptake corresponding to any cervical or thoracic lesion discriminable from thyroid tissue and positioned in typical parathyroid sites or in ectopic areas that were not typical lymph node sites, visible or not on CT images, was considered to be positive. For each lesion considered positive on PET/CT examination, maximum standardized uptake value (SUVmax), defined within a spherical volume of interest centered on the focal uptake and including it completely, metabolic tumoral volume (MTV), and Hounsfield units (HU) values were assessed. MTV was estimated using a fixed-threshold segmentation algorithm using a fixed-SUV threshold method (40% of SUVmax).

In patients with no contraindications and after a mutual agreement with the patient and the referring physician, a “one-stop” shop PET/4D-CT was performed after the intravenous injection of 75 mL of iomeprol (Iomeron 400, Bracco Imaging, Milan, Italy; 400 mg iodine/mL at 2.5–3 mL/s flow followed by a saline chaser) [30]. Four CT phases were successively acquired: non-contrast (140 kV, 115 mAs, 1s per rotation, pitch 0.8, slice thickness of 1 mm), arterial (10–15 s after injection, aortic arch threshold > 80 Hounsfield units), venous (45 s after injection), and late-venous phases (70 s after injection). CT parameters of the arterial and venous phases were as follows: 120 kV, 115 mAs, 1 s per rotation, pitch 0.8, and slice thickness of 1 mm. CT CARE Dose 4D combined with sinogram-affirmed iterative reconstruction (SAFIRE, Siemens Healthcare, Erlanger, Germany) was used. Diabetic patients withdrew metformin intake for 2 days after the 4D-CT scan, and abundant hydration was recommended. 4D-CT images were qualitatively interpreted as positive or negative by an expert head and neck radiologist, blinded to the results of other parathyroid imaging results. Any cervical or thoracic tissular lesion positioned in standard parathyroid sites or in ectopic areas with contrast-media enhancement and wash-out > 20 HU at late-venous phase, was considered as positive for hyperfunctioning parathyroid [14].

### 2.3. Gold Standard

Histological proof of hyperfunctioning parathyroid gland after parathyroidectomy was used as the gold standard for excised glands. Intraoperative parathyroid hormone monitoring was used to preserve the normal glands with resection of only the pathological(s) ones. Normal glands (true negative results) were defined according to a combination of neck surgical exploration, biological follow-up results (PTH, serum calcium values), and a fall of operative PTH serum concentration of more than 80% between pre-surgical dosage and approximately 120 min after parathyroidectomy. A PTH decrease of less than 70% suggested a surgical failure [31]. In the absence of intraoperative PTH monitoring information, a PTH measurement in the month following the operation was considered. In the case of persistent HPT, the remaining glands were deemed pathological.

### 2.4. Statistical Analysis

Results for continuous data were expressed as mean ± standard deviation (SD) or median and range as appropriate, whereas categorical variables were presented as numbers and percentages. The sensitivity, specificity, positive and negative predictive values (PPV and NPV) of neck US, 99mTc-sestamibi parathyroid scintigraphy, 4D-CT, and 18F-FCH PET/CT were calculated on a per lesion-based analysis. Diagnostic procedures were compared using the Chi2 or the Fisher’s exact test as appropriate. Nonparametric 2-tailed Mann Whitney *U* test was used for intergroup comparison. Correlations were assessed using the Spearman correlation test. Two-sided *p* values ≤ 0.05 were considered significant. Statistical analyses were performed using an open-access statistical software (biostatgv.sentiweb.fr, Institut Pierre Louis d’Epidémiologie et de Santé Publique, UMR S 1136, INSERM—Sorbonne Université, Paris, France).

## 3. Results

### 3.1. Patient Population

During the study period, 72 CKD patients with HPT were referred for 18F-FCH PET/CT. Among them, 30 patients were finally operated, and, thus, included in the study (14 women and 16 men, mean age: 55 years, range 23–73 years, 21 CKD G5 (including 18 in dialysis and 3 patients on kidney transplant waitlist treated by calcimimetic at time of imaging)), and 9 kidney transplant recipients. Eighteen (60%) had sHPT and 12 had tHPT.

The mean PTH level during the time of PET/CT was 1049 ng/L. Three patients were symptomatic, and three underwent neck surgery for thyroid-related diseases. Eleven patients had known benign thyroid diseases: nine with nodular goiter and two with Graves’ disease. Four patients (13%) presented with recurrent/persistent HPT, and the remaining 26 cases (87%) had no previous parathyroid surgery. In the whole studied population, the median interval between 18F-FCH PET/CT and parathyroidectomy was 130 days (range 15–460 days). However, 28 out of 30 patients (93%) were operated on less than 1 year after PET/CT: 126 days (15–320 days). The COVID pandemic was the main reason for the delay between imaging and surgery, reflecting the real-life conditions of our study. The patients’ characteristics are detailed in Table 1.

A total of 119 glands were considered in the analysis, and 74 were removed. Of the ones removes, 65 were hyperplasic, six were adenomatous, and three were normal. Bilateral neck exploration was performed in all cases. PTX with auto-transplantation in the forearm was performed in 2/30 patients. Due to a suspicion of synchronous thyroid cancer, thyroid lobectomy was performed in 3/30 patients, and total thyroidectomy in 4/30 patients. The resected glands were 16 upper right (22%), 19 lower right (26%), 16 upper left (22%), 20 lower left (27%), and three ectopic (4%): one alongside the thyro-thymic ligament, one in the upper mediastinum, and one in the right cervical localization. No intrathyroidal parathyroid was found. According to pathology report, the largest mean diameter of hyperfunctioning parathyroids was 1.54 ± 0.49 mm, the mean volume was 1.15 ± 1.2 mm^3^, and the mean weight was 0.69 ± 0.57 g.

### 3.2. Parathyroid Imaging

All patients had at least one preoperative imaging modality: 30 (100%) patients underwent 18F-FCH PET/CT, 22 (73%) had neck ultrasonography, 12 (40%) had 99mTc-sestamibi parathyroid scintigraphy, and 11 (37%) had neck 4D-CT, respectively. No pathological parathyroids (false negative study) were detected in three out of 30 patients (10%) by 18F-FCH PET/CT, in 10/22 (45%) by neck ultrasonography, in 2/12 (17%) by 99mTc-sestamibi parathyroid scintigraphy, and in 4/11 (36%) by neck 4D-CT.

Sensitivity, specificity, PPV, NPV, and global accuracy of each imaging modality on a per-lesion analysis are detailed in Table 2 and the results of a head-to-head comparison between 18F-FCH PET/CT and the other imaging techniques are reported in Table 3.

**Figure 1:**
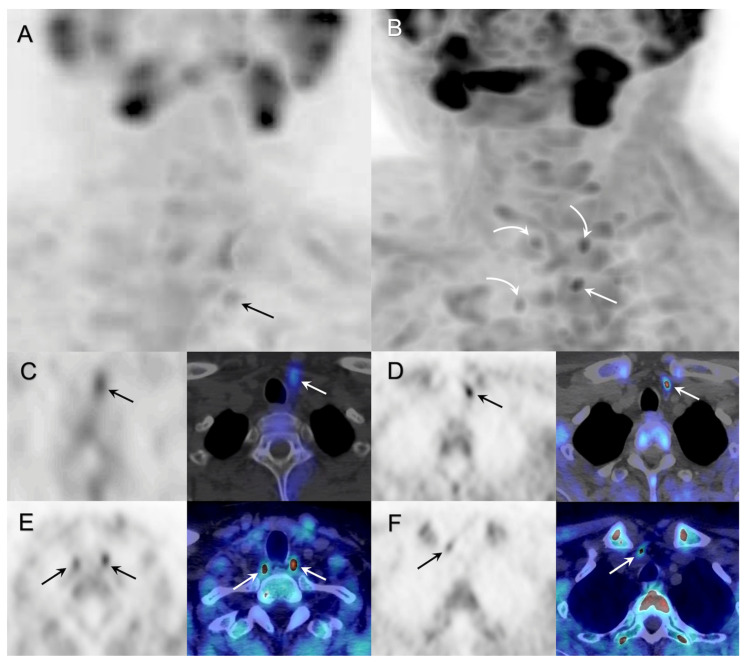
33-year-old kidney transplant recipient with tHPT. Cervical US was negative, and parathyroid scintigraphy (arrows, (**A**): MIP SPECT anterior image, (**C**): axial SPECT and SPECT/CT) suggested a hyperfunctioning left inferior parathyroid. Preoperative 18F-FCholine PET/CT showed increased focal uptake in all four parathyroids (arrows, (**B**): MIP PET anterior image, (**D**–**F**): axial PET and PET/CT). The patient underwent bilateral neck exploration as well as left superior and left inferior parathyroidectomy. Pathology showed parathyroid hyperplasia in both glands. The reduction of preoperative PTH serum concentration was 60%, and the patient presented with recurrent HPT about 12 months after surgery.

In the whole population, 18F-FCH PET/CT was significantly more sensitive and accurate (72%, 71%) than neck US (25%, 43%), 99mTc-sestamibi parathyroid scintigraphy (35%, 47%), and 4D-CT (40%, 47%) (Figure 1). Moreover, ^18^F-FCH PET/CT sensitivity was significantly higher in tHPT (88%) than in sHPT (66%). In an effort to explain the difference of 18F-FCH PET/CT sensitivity according to the type of HPT, we looked for a difference in glandular weight, volume, and size between sHPT and tHPT without finding statistically significant differences. The percentage of patients treated with calcimimetic was not significantly higher in sHPT (13/18, 72%) than in tHPT (7/12, 58%).

The specificity of 18F-FCH PET/CT (69%) was lower than that of neck US (95%) and of 99mTc-sestamibi parathyroid scintigraphy (90%) without reaching a statistical significance, probably due to the small size of the sample. Similar results in terms of diagnostic accuracy were found considering only the patients who underwent 18F-FCH PET/CT and neck US (*n* = 22, 72% vs. 43%, *p* < 0.001), or 99mTc-sestamibi parathyroid scintigraphy (*n* = 12, 72% vs. 47%, *p* = 0.01), or 4D-CT (*n* = 11, 65% vs. 47%, *p* = 0.08). Among the 11 patients who underwent integrated PET/4D-CT, 4D-CT found only one pathological gland not detected by 18F-FCH PET/CT. When analyzing the 10 patients explored by 18F-FCH PET/CT, neck US, and 99mTc-sestamibi parathyroid scintigraphy, the diagnostic accuracy of 18F-FCH PET/CT was higher than that of the other techniques: 68% vs. 33% (*p* = 0.002) vs. 48% (*p* = 0.07) (Table 4). The accuracy of 18F-FCH PET/CT was comparable between patients with sHPT and tHPT (77% vs. 68%, *p* = 0.3). However, 18F-FCH PET/CT was the most accurate technique in both patient groups.

According to a per lesion-based analysis performed on the overall population, 18F-FCH PET/CT was falsely positive in nine cases: two inflammatory lymph nodes, one papillary thyroid carcinoma, and six parathyroid glands judged normal in five patients during surgical neck exploration despite intense 18F-FCH uptake, and, thus, not resected. Of note, two of these five patients developed recurrent HPT two months after surgery, questioning whether hyperfunctioning but morphologically still normal glands were truly present at the time of the surgery. On the other hand, 22 (31%) pathological parathyroids (all hyperplasic glands) were not identified by 18F-FCH PET/CT. In three patients, with four proven parathyroid hyperplasia, 18F-FCH PET/CT was negative. Among them, three glands were detected by neck US, one gland by 99mTc-sestamibi parathyroid scintigraphy, and the other by 4D-CT.

In the whole population, the mean SUVmax and MTV of hyperfunctioning glands were 6.3 ± 3.4 (range: 1.6–18.7) and 2.9 ± 5.5 (range: 0.3–40.8), respectively. The mean weight and size of PET-positive glands were, respectively, 0.8 ± 1.1 g (range: 0.1–5.8 g) and 16 ± 6 mm (range: 9–35), and that of PET negative glands were 0.3 ± 0.3 g (range: 0.1–0.8 g) (*p* = 0.06) for the former and 13 ± 5 mm (range: 0.7–2.5) (*p* = 0.03) for the latter. Correlations between SUVmax, MTV, size, and weight have been investigated. Positive moderate significant correlations were found between weight and both SUVmax (r = 0.59, *p* = 0.00) and MTV (r = 0.56, *p* = 0.00).

Three hyperfunctioning ectopic parathyroids were diagnosed in three patients with sHPT. 18F-FCH PET/CT and 99mTc-sestamibi parathyroid scintigraphy were concordantly positive in two cases. Only one additional gland was detected by 18F-FCH PET/CT. US and 4D-CT failed to detect any ectopy in all three cases.

**Figure 2:**
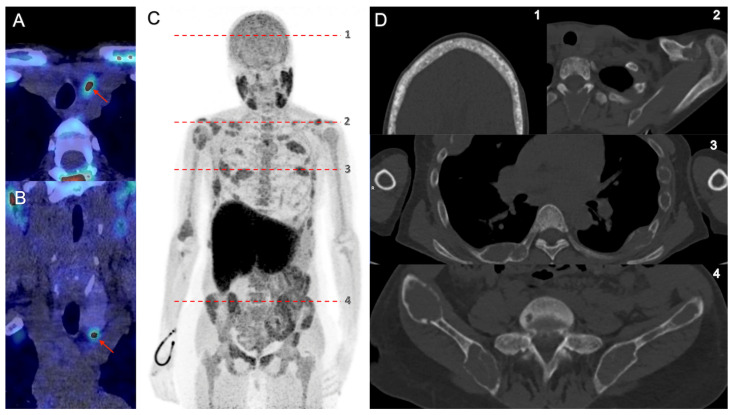
A 27-year-old woman with a history of kidney transplantation and hemodialysis was referred for sHPT. 18F-FCholine PET/CT revealed a left inferior hyperfunctioning parathyroid gland (arrows, (**A**): axial PET/CT, (**B**): coronal PET/CT) and multiples bones uptakes ((**C**): MIP PET anterior image) corresponding to both cystic lesions and patches of osteolysis (**D**) of the distal clavicles (**D.2**), a “salt and pepper” appearance of the skull (**D.1**), and both bone cysts and brown tumors in axial and extra-axial bones (**D.3–4**) allowing the diagnosis of osteitis fibrosa cystica (OFC). The patient was operated on, and pathology revealed a left inferior parathyroid hyperplasia.

In one patient with sHPT, an additional whole-body 18F-FCH PET/CT imaging was achieved after standard acquisition, showing multiple foci of skeletal uptake corresponding to both cystic lesions and patches of osteolysis in axial and extra-axial skeletal consisting of osteitis fibrosa cystica (Figure 2).

## 4. Discussion

The results of the present study confirm the diagnostic relevance of 18F-FCH PET/CT, which appears to be an effective imaging option for the preoperative detection of hyperfunctioning parathyroids, even in patients with CKD-related HPT. Our results contribute to the discussion on the optimization of the management of these patients. To the best of our knowledge, this is the largest clinical cohort examined by 18F-FCH PET/CT to this day, which also provides specific information on diagnostic performances in sHPT and tHPT, considered separately. 18F-FCH PET/CT outperformed neck US, 99mTc-sestamibi parathyroid scintigraphy, and contrast-enhanced CT in terms of diagnostic accuracy, also showing the highest sensitivity in both sHPT and tHPT patients. On the other hand, when we compared 18F-FCH PET/CT with the other diagnostic techniques, in particular neck US, 18F-FCH PET/CT had low specificity with higher rates of false positive results in patients with both sHPT and tHPT. These findings are consistent with the data previously reported by Xue et al. [27] and Chen et al. [28] in similar populations of CKD patients with HPT. A recent study revealed excellent results for PET/CT with 11C-Metionine (11C-MET) as a second-line preoperative technique after negative first-line ultrasound and scintigraphy in patients with tertiary hyperparathyroidism. In nine operated patients, the sensitivity of PET/CT with 11C-MET in the preoperative localization of patients with tHPT was 100%. Multiple lesions were visualized in 11 and ectopic lesions in four of the 19 studied patients [32].

Regardless of the choice of surgical procedure that will be performed (e.g., subtotal parathyroidectomy or total parathyroidectomy with auto transplantation), the identification of all hyperfunctioning parathyroid tissue may be required by the surgeon to prevent persistent/recurrent disease and to minimize the duration, as well as the number, of surgical procedures in this vulnerable population [33]. However, the role of pre-surgical imaging in CKD patients with HPT is probably different in patients with sHPT and tHPT and should be differentiated. While in sHPT all four glands can be considered as hyperplasic and hyperfunctioning, in tHPT it is often a single gland (or two) that develops an autonomic adenoma, a clinical situation similar to patients with pHPT. Indeed, the sensitivity and the NPV of 18F-FCH PET/CT in tHPT is higher than that found in sHPT, approaching the values published about patients with primary HPT [19]. In the aim of reducing extensive surgery, the accurate detection of pathological parathyroids may hold higher significance in tHPT patients rather than in patients with sHPT. In fact, because bilateral cervicotomy is often performed in sHPT, the role of preoperative imaging prior to initial surgery remains controversial [34]. In these patients, preoperative imaging can be useful for the identification of orthotopic enlarged parathyroid glands in the neck with distinction between thyroid nodules from nodular parathyroid hyperplasia, for the localization of ectopic and supernumerary parathyroid glands, and as guidance in the choice of the most appropriate parathyroid gland for preservation [35]. The detection of ectopic parathyroids is probably the most important contribution that preoperative imaging can make. Therefore, it is important to have a high-sensitivity pre-surgical imaging tool. In our study, 18F-FCH PET/CT found three ectopic glands, while two were found by scintigraphy and none by 4D-CT or ultrasound.

It is worth mentioning that the failure to identify the four parathyroid glands in sHPT usually results in persistent HPT after surgery [36,37]. In three sHPT patients (10%), PET did not detect parathyroid abnormalities, whereas the anatomopathological study revealed a hyperplasia of all four glands. Correlations between SUVmax, MTV, size, and weight have been investigated, and only moderate significant correlation has been found between weight and both SUVmax (r = 0.59), and MTV (r = 0.56). Glands weighting more than 1 g were always detected by 18F-FCH PET/CT. On a physio-pathological standpoint, an increased gland volume may suggest monoclonal rather than polyclonal proliferation, indicating an autonomous parathyroid and a more advanced stage of the disease. For this purpose, preoperative imaging showing an enlarged gland with high SUVmax and MTV can be of interest where nodular transformation is known to be less responsive to medical therapy than diffuse hyperplasia [38]. On the other hand, the assessment of metabolic indexes as SUVmax and MTV could allow the selection of the glands to be removed in priority when the choice has been made to leave one or half of a gland in place to avoid postoperative hypocalcemia. Prospective investigations are needed before considering 18F-FCH PET/CT to guide the surgical procedure and glandular sparing.

**Figure 3:**
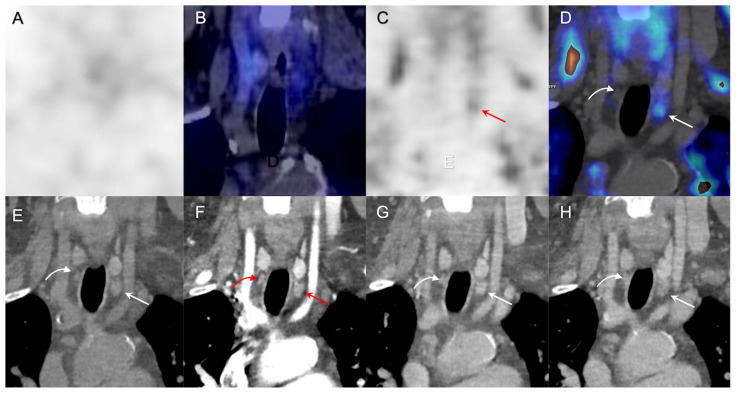
18F-FCH PET/CT and 4D-CT results in a 60-year-old woman with a history of kidney transplantation and dialysis referred for a secondary hyperparathyroidism (PTH: 775 ng/L, calcemia: 2.45 mmol/L). From left to right: coronal SPECT (**A**), coronal SPECT/CT (**B**), coronal PET (**C**), coronal PET/CT (**D**), non-contrast-enhanced CT (**E**), arterial-phase CT (30 s after injection) (**F**), venous-phase CT (45 s after injection) (**G**), and late-venous phases (70 s after injection) (**H**). Preoperative parathyroid scintigraphy and US failed to detect hyperfunctioning gland, and 18F-FCholine PET/CT showed increased focal uptake only in the left inferior parathyroid (arrow). 4D-CT revealed an additional right inferior pathological parathyroid (curbed arrow). The patient underwent bilateral neck exploration, and both left and right inferior parathyroidectomy. Pathology showed parathyroid hyperplasia in both glands. Reduction of preoperative PTH-serum concentration was greater than 80%. The patient showed a normal biological profile during her 12 months post-surgical follow-up.

A combination of morphological and molecular techniques is generally recommended in HPT patients as a first-line preoperative imaging work-up, but no consensus has been reached concerning CKD patients and the best choice and sequence for optimization of patient management. In a retrospective review of 40 patients with sHPT and tHPT, Pham et al. [33] underlined the limits of 99mTc-sestamibi parathyroid scintigraphy to detect multiglandular disease (sensitivity: 27%), especially for tiny hyperfunctioning hyperplasic parathyroid tissue. A diagnostic improvement could be reached when scintigraphy is coupled with CT in SPECT/CT studies. In a group of 109 patients with sHPT investigated preoperatively, US had the highest sensitivity (91.5%) and 99mTc-sestamibi parathyroid scintigraphy (56.1%) had the lowest [39]. The sensitivity of combined US and contrast-enhanced CT (95.0%) was the highest among the combination of two modalities. More recently, a combined 18F-FCH PET/4D-CT examination has been challenged in patients with primary HPT, potentially taking advantage of the superior spatial/temporal resolution of CT as well as the high sensitivity of the metabolic PET examination [30,40,41]. However, the real diagnostic advantages of such an approach are not entirely clear. Indeed, a recent head-to-head comparison identified only a slight difference in sensitivity when the combined examinations were used. Nevertheless, its impact on sensitivity is still a matter of debate, and the combined use of 18F-FCH PET and 4D/CT in one-stop-shop examination could potentially help with the differential diagnosis, lowering the incidence of false positive PET/CT results (i.e., inflammatory lymph nodes, thyroid nodules) and improving specificity and positive predictive value. The 4D-CT could be integrated in case of inconclusive or unclear findings on the PET examination. These situations include small adenomas, localizations close to the thyroid, and glands with a relevant cystic component. However, the use of the combined methods increases the patient’s radiation exposure, requires expertise, and introduces a slight risk due to the contrast medium administration when compared with 18F-FCH PET/CT. Furthermore, the administration of CT contrast media could often be contraindicated in patients with HPT and CKD, reducing the potential utilization of 4D-CT. In our study, 4D-CT revealed only one additional pathological gland not detected by 18F-FCH PET in 11 patients who underwent integrated PET/4D-CT (Figure 3).

Although in patients with HPT, 18F-FCH PET/CT is usually focused on the neck and mediastinum, whole-body examination could be performed, allowing the detection of synchronous HPT-related metabolic alterations as brown tumors [42].

In one patient with sHPT, whole-body 18F-FCH PET/CT revealed axial and extra-axial skeletal alterations consisting in osteitis fibrosa cystica (OFC) (Figure 3). OFC represents a serious complication of kidney osteodystrophy, predominantly affecting the hands, feet, skull, and facial bones. OFC is a bone disorder caused by severe hyperparathyroidism and resulting in the formation of subperiosteal bone resorption, osteolysis of the distal clavicles, a “salt and pepper” appearance of the skull, bone cysts, and brown tumors of the bones. After parathyroidectomy, patients usually show complete healing of subperiosteal resorption, partial repair of bone cysts, and their cortical bone mineral content might increase. Nevertheless, despite the improvement of both imaging techniques and laboratory methods for early detection of hypercalcemia making OFC a rare complication in developed countries, it should be investigated because of its high impact on mortality and morbidity of CKD patients with HPT.

Our study suffers from several limitations. First, the inclusion of a relatively limited number of patients. Indeed, among the 72 CKD patients with HPT referred for preoperative 18F-FCH PET/CT during the study period, only 30 patients were finally operated on and included in the study. Some patients’ refusal of surgery and the postponing of some surgeries due to the COVID-19 pandemic explains the low rate of patients who underwent surgery. However, the cohort studied represents the largest population examined by 18F-FCH PET/CT. Second, mainly due to the retrospective nature of the study, neck US, parathyroid scintigraphy, and 4D-CT were not performed in all patients, precluding robust comparison among diagnostic techniques and potentially introducing a patient selection bias. However, in our tertiary referral surgical center, we faced an important variability in the diagnostic precision of the imaging studies that patients had already obtained before their referral. Our practice is to avoid additional and potentially unnecessary exposition to radiation, additional costs, and delays. Finally, the relatively long interval between imaging and parathyroidectomy should also be considered as a limitation of our study, as it may increase the possibility of false negative results (a long interval may be responsible for pathological evolution of gland secretion and volume).

## 5. Conclusions

The role of preoperative imaging for localization hyperfunctioning parathyroids is debated in CKD patients with HPT. Hence, the need to know the spectrum of their diagnostic performances and inherent limitations. Cervical US and 99mTc-sestamibi scintigraphy have a good specificity but too low of a sensitivity to detect multiglandular diseases. 4D-CT does not provide sufficient sensitivity, and it is often contraindicated for patients with kidney failure. 18F-FCH PET/CT appears to be an effective imaging option in patients with sHPT and tHPT, and had better results than cervical US, 99mTc-sestamibi scintigraphy, and 4D-CT. To reduce extensive surgery, the accurate detection of pathologic parathyroids is of greater importance in patients with tHPT (who could benefit from minimally invasive parathyroidectomy) than in patients with sHPT, who often undergo bilateral cervicotomy. In these cases, preoperative 18F-FCH PET/CT may be helpful in locating ectopic glands and may guide the surgical choice for gland preservation to avoid postoperative hypocalcemia. In patients with tHPT, 18F-FCH PET/CT could be proposed as preoperative imaging, followed by a cervical ultrasound in case of a doubtful positive localization, to disregard nodal false positive results. The surgical impact of these findings should be assessed in prospective multicenter clinical trials.

## Figures and Tables

**Table 1:** Patient characteristics and mineral disorders before parathyroidectomy. sHPT: secondary hyperparathyroidism, tHPT: tertiary hyperparathyroidism.

sHPT/tHPT	18/12	
Sex	14F/16M	
Age (y)	55 ± 27	(range, 23–73)
Calcemia (mmol/L) (mean ± SD)	2.37 ± 0.28	(range, 1.54–3.15)
Phosphorus (mmol/L) (mean ± SD)	1.2 ± 0.55	(range, 0.67–2.44)
25-OH-Vitamin D (ng/mL) (mean ± SD)	31.9 ± 15.4	(range, 8.5–49.8)
PTH (ng/L) (median)		
Whole population	1049.4 ± 830	(range, 69.1 *–3178)
CKD G5 (21 patients, 18 dialyzed)	1205	(range, 95–3178)
Transplanted patients (2 CKD G2T, 7 CKD G3T)	278	(range, 69.1–699)
Treatment	21/30	20 calcimimetic; 1 calcitriol/vitamin D analogs
Dialysis	18/30	14 sHPT/4 tHPT
Kidney transplant	9/30	9 tHPT
CKD G5 not on dialysis (transplant waitlist)	3/30	3 sHPT
Complications	5/30	Osteopenia (3/30); conduction disorders (1/30); brown tumors and fibrocystic osteitis (1/30)
Thyroid disease	11/30	Nodular (9/30); Graves’ disease (2/30)
Time interval 18F-FCH and surgery (d) (median)	130	(range 15–460 days)

*: Patient with PTH of 69.1 ng/L was transplanted patient with hypercalcemic HPT treated with calcimimetic (tHPT).

**Table 2:** Diagnostic performances (per lesion-based analysis) of 18F-FCH PET/CT, neck US, 99mTc-sestamibi parathyroid scintigraphy, and 4D-CT. sHPT: secondary hyperparathyroidism, tHPT: tertiary hyperparathyroidism. TP: true positive, TN: true negative, FP: false positive, FN: false negative, Se: sensitivity, Sp: specificity, PPV: positive predictive value, NPV: negative predictive value.

Imaging Modality	All Patients	Glands	TP	TN	FP	FN	Se	Sp	PPV	NPV	Accuracy
PET/CT	30	119	65	20	9	25	72%	69%	88%	44%	71%
Cervical US	22	87	17	18	1	51	25%	95%	94%	26%	43%
^99m^Tc-sestamibi scintigraphy	12	47	13	9	1	24	35%	90%	93%	27%	47%
4D-CT	11	43	14	6	2	21	40%	75%	88%	22%	47%
	**sHPT patients**	**Glands**	**TP**	**TN**	**FP**	**FN**	**Se**	**Sp**	**PPV**	**NPV**	**Accuracy**
PET/CT	18	71	42	6	1	22	66%	86%	98%	21%	68%
Cervical US	14	55	11	5	1	38	22%	83%	92%	12%	29%
^99m^Tc-sestamibi scintigraphy	10	38	11	6	0	21	34%	100%	100%	22%	45%
4D-CT	8	31	13	3	0	15	46%	100%	100%	17%	52%
	**tHPT patients**	**Glands**	**TP**	**TN**	**FP**	**FN**	**Se**	**Sp**	**PPV**	**NPV**	**Accuracy**
PET/CT	12	48	23	14	8	3	88%	64%	74%	82%	77%
Cervical US	8	32	6	13	0	13	32%	100%	100%	50%	59%
^99m^Tc-sestamibi scintigraphy	2	9	2	3	1	3	40%	75%	67%	50%	56%
4D-CT	3	12	1	3	2	6	14%	60%	33%	33%	33%

**Table 3:** Head-to-head comparison (*per lesion*-based analysis) between ^18^F-FCH PET/CT, neck US, ^99m^Tc-sestamibi parathyroid scintigraphy, and 4D-CT.

	PET/CT vs. Cervical US	PET/CT vs. Scintigraphy	PET/CT vs. 4D-CT
Sensitivity	*p* < 0.001	*p* < 0.001	*p* < 0.001
Specificity	*p* = 0.07	*p* = 0.4	*p* = 1
Positive Predictive Value	*p* = 0.7	*p* = 1	*p* = 1
Negative Predictive Value	*p* = 0.04	*p* = 0.1	*p* = 0.06
Accuracy	*p* < 0.001	*p* = 0.003	*p* = 0.003

**Table 4:** Head-to-head comparison (*per lesion*-based analysis) between ^18^F-FCH PET/CT, neck ultrasonography, ^99m^Tc-sestamibi parathyroid scintigraphy, and contrast-enhanced CT in patient population.

Performed Imaging	Patients	Glands	TP	TN	FP	FN	Se	Sp	PPV	NPV	Accuracy (*p*)
PET/CT, scintigraphy	12	47	28	6	4	9	76%	60%	88%	40%	72% (*p* = 0.01)
			13	9	1	24	35%	90%	93%	27%	47%
PET/CT, 4D-CT	11	43	23	5	3	12	66%	63%	88%	29%	65% (*p* = 0.08)
			14	6	2	21	40%	75%	88%	22%	47%
PET/CT, US	22	87	49	14	7	18	73%	67%	88%	44%	72% (*p* < 0.001)
			17	18	1	51	25%	95%	94%	26%	43%
PET/CT, US, scintigraphy	10	40	22	5	4	9	71%	56%	85%	36%	68% (*p* = 0.007)
			5	8	1	26	16%	89%	83%	24%	33%
			11	8	1	20	35%	89%	92%	29%	48%

## Data Availability

Purely observational studies do not require registration.
